# Integrative evolutionary and structural modeling of leaf rust resistance genes in wheat (*Triticum aestivum* subsp. and *T. monococcum*)

**DOI:** 10.3389/fpls.2026.1867181

**Published:** 2026-08-05

**Authors:** Meshal Munawir R. Almutairi

**Affiliations:** King Abdul-Aziz City for Science and Technology, Advanced Agricultural and Food Technology Institute, Riyadh, Saudi Arabia

**Keywords:** (*Triticum aestivum* subsp), canonical ABC transporter, motif analysis, phylogenetic tree, short for leaf rust resistance gene 34 (Lr34)

## Abstract

This study investigated the evolutionary relationships and molecular characteristics of selected genes in wheat (*Triticum aestivum subsp*.) using phylogenetic reconstruction and motif analysis. A phylogenetic tree was constructed using the Maximum Likelihood method, and conserved motif patterns were identified using ClustalW alignment and R-based analyses. The detection of a shared conserved motif across genes G3 to G11 at the same position suggests functional conservation, shared regulatory roles, or evolutionary constraint. Highly significant motifs identified in Gen1 and Gen2 indicate potential functional importance, possibly corresponding to active or binding sites. Phylogenetic analysis grouped the genes into two main clusters; a strongly supported monophyletic clade (Gen1, Gen10) suggests conserved functional roles, whereas weaker support among Gen13–Gen18 limits resolution of deeper evolutionary relationships. Sequence logo analysis revealed conserved regions between positions 107 and 301, particularly at residues P, L, R, F, G, and K, while positions 200–260 exhibited low conservation, indicating potential functional divergence. Structural prediction using AlphaFold2 revealed a canonical ABC transporter fold with high-confidence nucleotide-binding domains and a membrane-spanning helical bundle containing conserved catalytic residues, supporting an ATP-dependent transport function in wheat. Overall, these findings provide insights into the evolutionary history, structural conservation, and potential functional roles of these genes in wheat.

## Introduction

Cereals, particularly wheat (*Triticum aestivum subsp*. and *T. monococcum*), are a cornerstone of global food security. With a rapidly growing population and increasing climate variability, there is an urgent need to develop wheat varieties that can withstand multiple pests and diseases while maintaining high grain yield and quality (Zhang et al., 2025; Tilman et al., 2011).

Among the major biotic threats affecting wheat, leaf rust is particularly significant due to its widespread occurrence across diverse agroecological regions and its capacity to cause substantial yield losses. In this context, resistance genes have emerged as an effective and sustainable strategy for disease management. Among these, the Lr34 gene is notable for conferring durable and broad-spectrum resistance, making it a key target for evolutionary, genomic, and breeding studies (Zhang et al., 2024; Michel and Makowski, 2013).

The *Lr34* gene has been recognized for over a century as being associated with durable, partial resistance to multiple wheat diseases, including leaf rust, stem rust, stripe (yellow) rust, and powdery mildew. A key feature of this gene is its ability to confer consistent and long-lasting resistance across seasons and environments. This contrasts with many major (race-specific) resistance genes, which are often overcome rapidly by the evolution of new pathogen races (Ellis et al., 2014).

The resistance conferred by *Lr34* is frequently associated with a characteristic phenotype known as leaf tip necrosis (LTN). This trait is widely used as a morphological marker in breeding programs, as it is often correlated with the presence of durable resistance across diverse genetic backgrounds (Krattinger et al., 2009).

The evolutionary characterization of the *Lr34* gene remains insufficiently understood. However, population genetics and genomic studies have revealed notable allelic variation at the *Lr34* locus between traditional wheat landraces and modern cultivated varieties. In addition, differences in allele frequencies have been reported across diverse geographical regions (Babu et al., 2020).

This observed diversity is likely shaped by multiple factors, including historical variation in landrace genetic pools, farmer-driven selection practices, modern breeding programs prioritizing other agronomic traits, and regional adaptation to local environmental conditions and pathogen pressures (Singh et al., 1998).

Moreover, the haplotype structure surrounding *Lr34* may provide evidence of introgression or gene flow from other *Triticum* species or wild relatives, suggesting the potential acquisition of novel or unexpected resistance alleles within specific gene pools (Li et al., 2023).

An additional perspective integrating evolutionary biology and functional genomics focuses on the relationship between nucleotide variation in *Lr34* and its effects on gene regulation and expression. It is likely that resistance is not solely driven by changes in the coding sequence, but may also involve variation in regulatory elements that influence expression levels or alter protein structure and function. Early studies have shown that *Lr34* is expressed in mature flag leaves and is associated with upregulation under abiotic stress conditions (Shah et al., 2010, 2011).

Accordingly, comparative sequence analyses, dN/dS ratio tests across phylogenetic lineages, and the characterization of regulatory binding sites are essential approaches for elucidating the functional evolutionary history of this gene (Feuillet et al., 2003; Karelova et al., 2011; Krattinger et al., 2013).

From an applied perspective, understanding the evolutionary signature of *Lr34* is as important as its theoretical implications. If resistant alleles are shown to have an ancient and stable origin within certain populations, breeding programs can exploit them with minimal risk of unintended genetic effects. Conversely, if resistance alleles are geographically restricted or associated with deleterious linkage drag, more targeted strategies may be required, such as fine mapping, gene editing to introduce optimized alleles into elite backgrounds, or the development of precise molecular markers for marker-assisted selection (Dieguez et al., 2014).

Furthermore, elucidating genetic interactions (epistasis) between *Lr34* and other resistance loci is critical to ensure that pyramiding strategies produce additive or synergistic effects rather than antagonistic interactions that could compromise resistance durability (Cloutier et al., 2023; Hewitt et al., 2021; Yaniv et al., 2015; Hulbert et al., 2007).

The present study aimed to conduct an evolutionary and molecular characterization of the wheat leaf rust resistance gene Lr34 by analyzing nucleotide and amino acid sequence variation across diverse Triticum aestivum genotypes. Specifically, it seeks to elucidate the evolutionary dynamics and functional implications of allelic diversity at the Lr34 locus through the application of integrative bioinformatics and statistical approaches.

## Materials and methods

Sequences related to *Lr34* in wheat were retrieved using FASTA-based searches against the genomes of allohexaploid wheat (*Triticum aestivum* subsp. *aestivum*, AABBDD genome) and the diploid species *T. monococcum* subsp. *aegilopoides* (einkorn wheat). **Identified** genes included members of the ABCG transporter subfamily, lectin receptor kinases, cytochrome P450s proteins, and hexose transporters. Candidate genes were selected based on sequence similarity to Lr34 and published evidence linking them to disease-resistance pathways. The study therefore includes both Lr34-related ABC transporters and a broader group of disease-resistance-associated genes involved in pathogen recognition, transport processes, signaling, and stress responses (**Table 1**).

**Table 1:** The list of Lr34 sequences used with their accession numbers according to NCBI database in *riticum aestivum* and *T. monococcum subsp. aegilopoides*.

No. Gen	Classification	Accession no.	Key domains identified
Gene1	ABCG/PDR transporter	ADK62371.1	NBD + TMD (12+ helices), ABC motifs
Gene2	ABCG/PDR transporter	ACN41354.1	NBD + TMD (12+ helices), ABC motifs
Gene3	ABCG transporter (TMD fragment)	AOC59466.1	TM helices
Gene4	ABCG transporter (TMD fragment)	AOC59465.1	TM helices
Gene5	ABCG transporter (TMD fragment)	AOC59464.1	TM helices
Gene6	ABCG transporter (TMD fragment)	AOC59463.1	TM helices
Gene7	ABCG transporter (TMD fragment)	AOC59462.1	TM helices
Gene8	ABCG transporter (TMD fragment)	AOC59461.1	TM helices
Gene9	ABCG transporter (TMD fragment)	AOC59460.1	TM helices
Gene10	ABCG transporter (TMD fragment)	AOC59459.1	TM helices
Gene11	ABCG transporter (TMD fragment)	AOC59458.1	TM helices
Gene12	Lectin receptor-like kinase (LecRLK)	ADK62373.1	Lectin + TM + Kinase
Gene13	Cytochrome P450	ADK62372.1	P450 motifs
Gene14	Hexose transporter (MFS)	ADK62370.1	MFS signatures, 12 TM
Gene15	Hexose transporter (MFS)	ADK62367.1	MFS signatures
Gene16	Cytochrome P450	ADK62366.1	P450 motifs
Gene17	Lectin receptor kinase (LecRLK)	ADK62369.1	Lectin + TM + Kinase
Gene18	Cytochrome P450	ADK62368.1	heme-binding site

These genes are associated with distinct disease resistance mechanisms in wheat. For instance, lectin receptor kinases have been implicated in resistance to stripe rust, cytochrome P450 enzymes are associated with Fusarium head blight resistance, and hexose transporters may contribute to pathogen resistance through interference with glucose uptake via a dominant-negative mechanism. Both coding and non-coding regions (introns and exons) were included in the dataset to capture comprehensive sequence variation.

Evolutionary relationships among the genes were inferred using multiple sequence alignment performed with the ClustalW algorithm in MEGA v12. Protein sequences were aligned using default parameters, and regions containing insertions and deletions were removed prior to phylogenetic reconstruction to improve alignment quality. Insertions and deletions were removed prior to analysis to improve alignment reliability. Pairwise genetic distances were calculated using the Kimura 2-parameter model, and phylogenetic reconstruction was performed using the maximum likelihood approaches to resolve evolutionary relationships among gene families. The final phylogenetic tree was generated using the Maximum Likelihood method with the Jones–Taylor–Thornton (JTT) amino acid substitution model. Branch support was evaluated using 1000 bootstrap replicates.

In parallel, motif analysis was conducted to identify conserved regions with statistical significance. The analysis was implemented in R (version 4.5.2) using RStudio, where a data frame (“motif”) was generated containing gene identifiers and motif start and end positions (**Table 2**). Motifs were classified based on combined p-values, with significance defined as p < 0.0001. This threshold was used to distinguish highly significant conserved motifs from non-significant patterns, allowing identification of functionally relevant sequence features across the gene set.

**Table 2:** Start and end position of identified motifs for Lr34 genes and their corresponding p-value and combined p-value.

Gene	Motif start	Motif end	p-value	Combined p-value
Gen1	442	906	1.09e-59	1.47e-56
Gen2	445	906	1.71e-57	2.31e-54
Gen3	3	149	4.82e-63	7.37e-61
Gen4	6	149	4.82e-63	7.52e-61
Gen5	6	149	4.82e-63	7.52e-61
Gen6	6	149	4.82e-63	7.52e-61
Gen7	6	149	4.82e-63	7.52e-61
Gen8	6	149	4.82e-63	7.52e-61
Gen9	6	149	4.82e-63	7.52e-61
Gen10	6	149	4.82e-63	7.52e-61
Gen11	6	149	4.82e-63	7.52e-61
Gen12	666	–	–	2.91e-01
Gen13	515	–	–	2.61e-01
Gen14	504	–	–	1.38e-01
Gen15	514	–	–	1.41e-01
Gen16	139	–	–	1.10e-01
Gen17	262	–	–	7.31e-01
Gen18	261	–	–	3.17e-02

Conserved protein motifs and domains were examined directly from sequence alignments to provide additional validation of functional annotations. Particular attention was given to the identification of characteristic ATP-binding cassette (ABC) transporter signatures, including Walker A/P-loop ATP-binding motifs, ABC signature motifs (LSGGQ), nucleotide-binding domains (NBDs), and hydrophobic transmembrane regions. Conserved motifs were compared with previously characterized ABC transporter proteins to evaluate consistency with the predicted transporter architecture.

## Results

**Figure 1:**
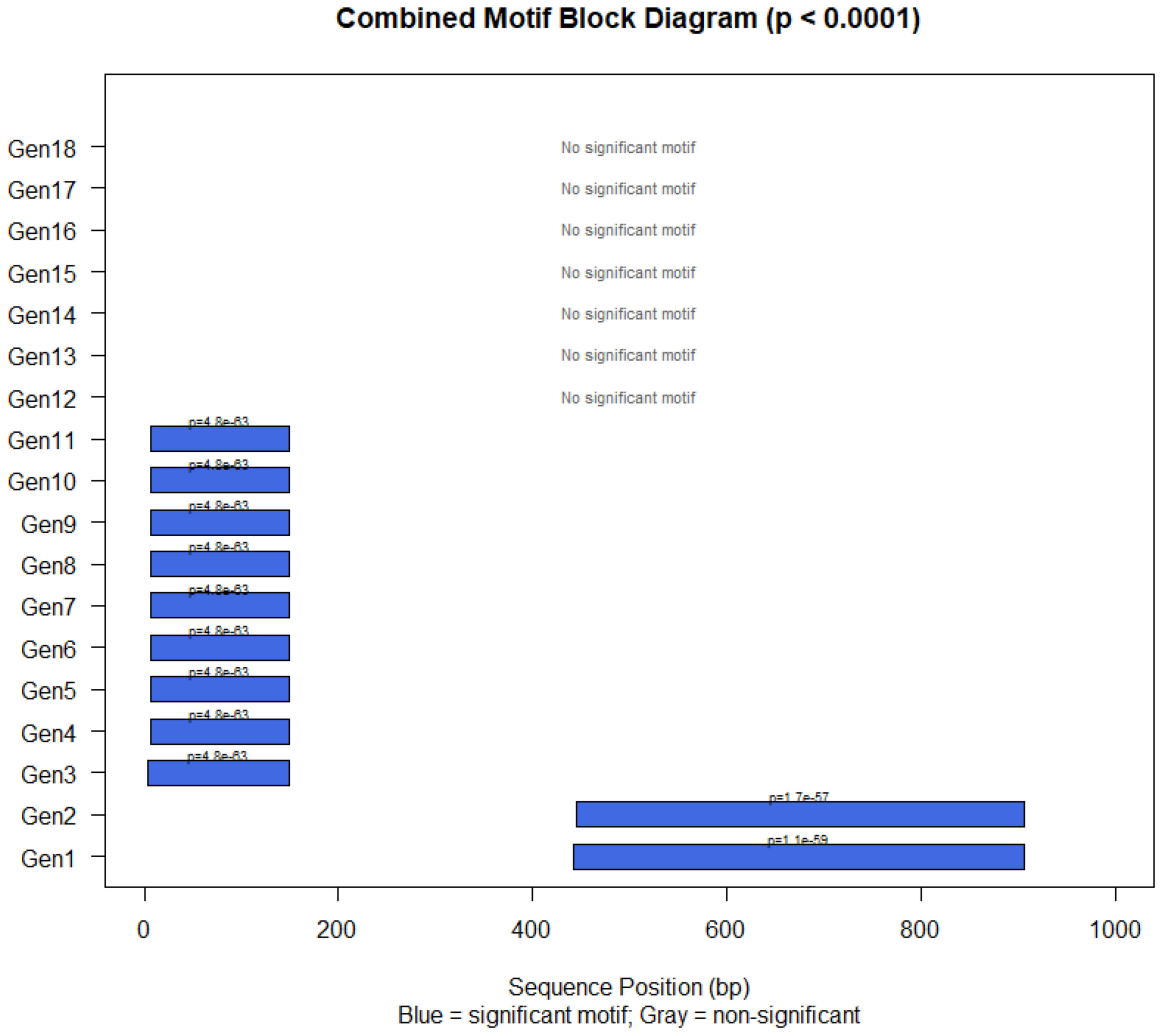
Combined motif architecture in wheat proteins showing statistically significant motifs (p < 0.0001), with amino acid position distribution and gene frequency representation.

The combined motif diagram was analyzed using R to enhance motif detection and to assess statistical significance (**Figure 1**). Gene1 to Gene11 showed a highly significant motif signal (p < 0.0001), whereas Genes12 to 18 did not exhibit statistically significant motifs. The strongest motif enrichment was observed in Gene1 and Gene2 (p = 1e−59 and 1e−57, respectively), located at approximately 450–950 bp. In contrast, Gene3 to Gene11 shared a conserved motif positioned at ~100 bp with a moderate significance level (p = 8e−03). The presence of highly significant motifs in Gene1 and Gene2 suggests functionally important putative or conserved predicted. Similarly, the shared motif among Gene3 to Gene11 indicates functional similarity and/or shared regulatory control, consistent with membership in a common gene family. In contrast, the absence of significant motifs in Gene12 to Gene18 suggests limited sequence conservation and a lack of statistically supported functional similarity within these sequences.

**Figure 2:**
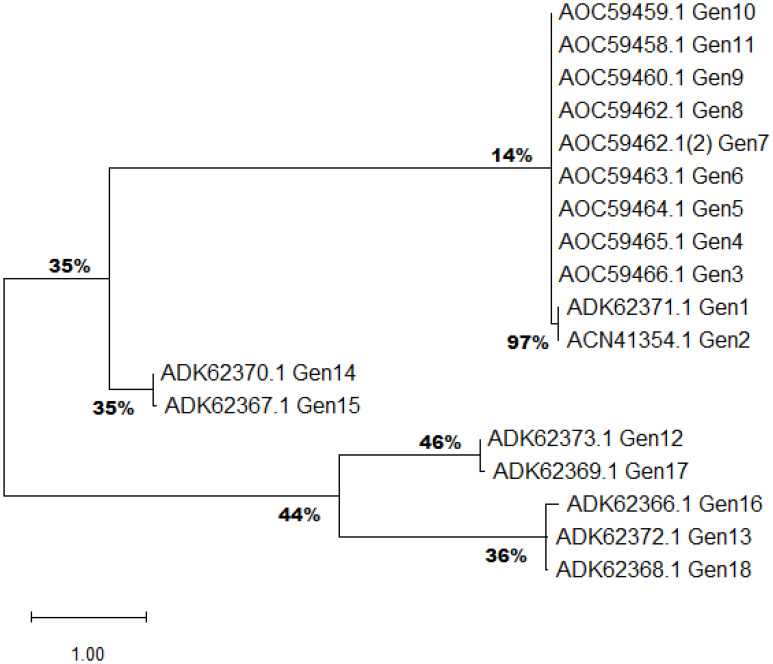
Maximum likelihood phylogenetic analysis of Lr34 protein sequences in wheat (18 amino acid sequences, 1,447 aligned positions) with bootstrap support and sequence logo visualization of conserved residues (MEGA12, parallel computing implementation).

A phylogenetic tree (phylogram/cladogram) was constructed based on Lr34 gene sequences from the wheat genome using the Maximum Likelihood (ML) method (**Figure 2**). The analysis employed the Jones et al. substitution model, a widely used amino acid replacement model for protein sequence data. The final model achieved a log-likelihood score of approximately −9,766.80. The dataset comprised 18 amino acid sequences with a total of 1,442 aligned positions, providing sufficient resolution and analytical robustness under the stated model assumptions.

The terminal nodes of the tree are positioned on the right side and labeled with gene names and accession numbers. The phylogeny is divided into several major clades. The first cluster, comprising Gene1 and Gene2, showed high support with 97% bootstrap value, indicating strong confidence in this grouping. The second cluster, including Gene3 to Gene11, exhibited low support (14%), suggesting weak phylogenetic signal. Additional groupings include Gene4 with Gene5 (35% support), Gene12 to Gene17 (46% support), and a final cluster comprising Genes13, 16, and 18 (36% support).

Bootstrap values were used to assess branch reliability, where values above 45% indicate moderate to strong support, whereas values below this threshold reflect weak support. Accordingly, the grouping of Gene3 to Gene11 (AOC5945x) shows insufficient phylogenetic resolution and may represent poorly resolved or stochastic relationships rather than strongly supported evolutionary divergence. Similarly, the ADK62xxx cluster is weakly resolved, with several internal nodes supported between 35% and 46%, indicating limited phylogenetic signal. This pattern may result from low sequence variation at the selected sites, rapid evolutionary divergence, or conflicting evolutionary signals such as recombination or accelerated mutation rates in specific lineages.

In addition, variation in branch lengths was observed across the tree. Branch length represents the estimated number of evolutionary substitutions. Short branches indicate limited sequence divergence and likely recent evolutionary separation, whereas longer branches suggest greater genetic divergence due to either older divergence events or higher evolutionary rates.

**Figure 3:**
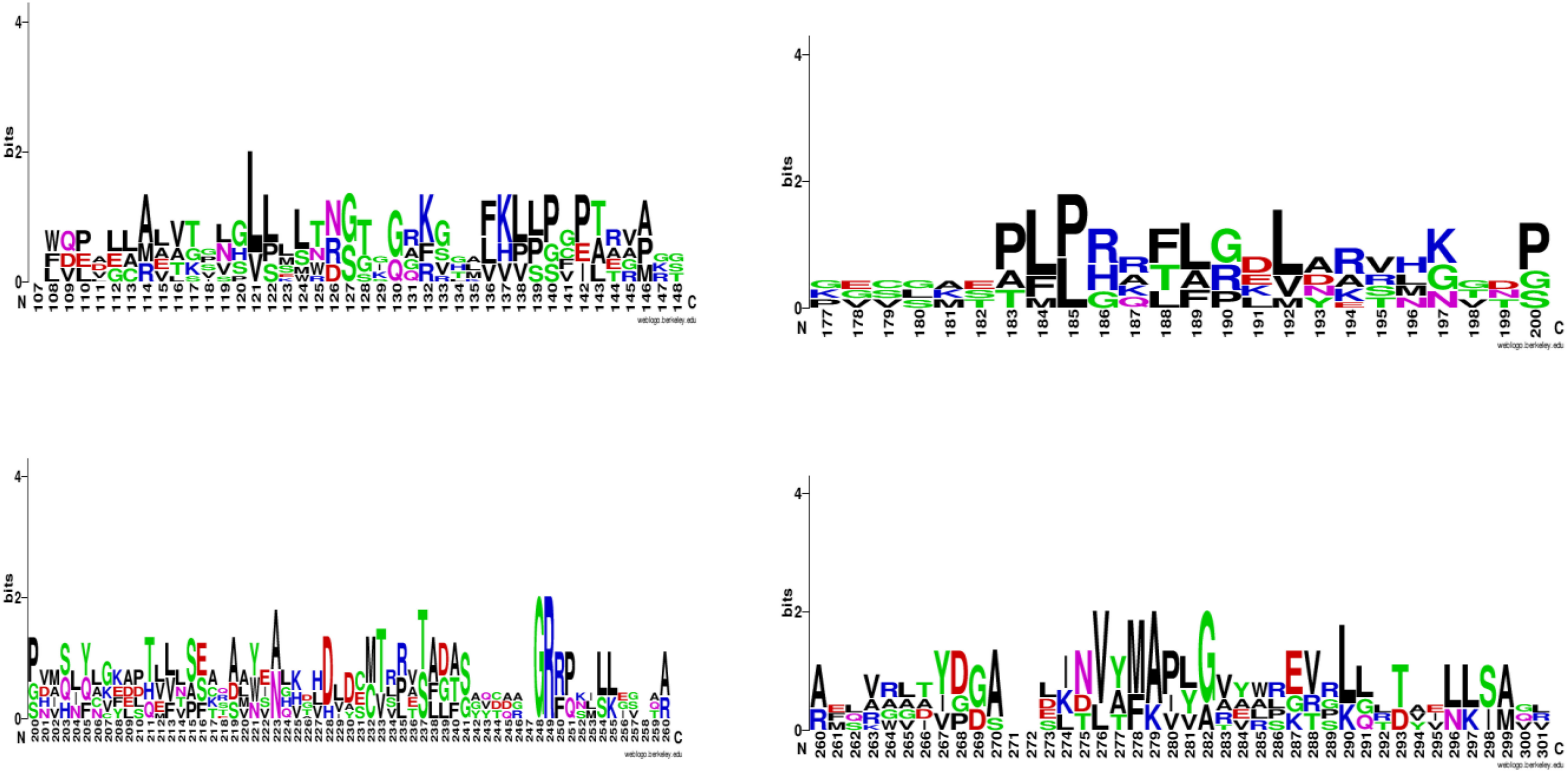
Sequence logo of aligned protein sequences (positions 107–301), where the horizontal axis represents amino acid positions and the vertical axis indicates information content (bits) reflecting positional conservation. Letter height corresponds to the degree of conservation at each position across all sequences, while amino acid composition and color variation reflect sequence variability.

The sequence logo was used to identify conserved functional residues and variable regions across the aligned protein sequences (positions 107–301) (**Figure 3**). Overall, the analysis revealed clear spatial patterns of evolutionary constraint and divergence within the examined segment.

A highly conserved region was observed between positions 107–200, characterized by strong sequence conservation with amino acids such as P, L, R, F, G, and K, and information content exceeding 3 bits at several positions. The presence of conserved short motifs, including PLP and FLG, suggests functional importance, as such patterns are commonly associated with putative catalytic or structurally constrained protein regions. This high level of conservation is consistent with strong evolutionary constraint and supports the close phylogenetic relationships observed among sequences clustering with high bootstrap support. Amino acids such as lysine (K), glycine (G), and phenylalanine (F) likely contribute to structural stability and putative functional roles related to protein folding, structural maintenance, and potential molecular interactions.

In contrast, the region spanning positions 200–260 exhibited pronounced sequence variability, as indicated by low information content and small, heterogeneous logo symbols. This suggests relaxed selective constraints and reduced functional importance. Such variability likely contributes to the low bootstrap support values observed in certain phylogenetic branches (3544%), reflecting increased uncertainty in evolutionary relationships due to sequence divergence in this region.

The segment from positions 260–301 showed an intermediate pattern, with a combination of conserved residues (e.g., Y, D, G, A, M, P, and L) and variable positions. This indicates a mixture of structural constraint and localized flexibility, suggesting that this region may include functionally important residues embedded within more tolerant sequence contexts.

From a structural perspective, conserved residues such as proline, lysine, arginine, phenylalanine, and glycine are typically associated with stabilization of secondary structure, loop formation, protein core integrity, and potential interaction sites with nucleic acids or other molecules. In contrast, poorly conserved regions are likely to correspond to surface exposed loops or flexible segments that contribute to functional diversification and species-specific adaptation.

Notably, highly supported phylogenetic nodes (up to 97%) correspond to sequences exhibiting greater conservation across these regions, particularly within the 260–301 segment, supporting their close evolutionary relatedness. Conversely, low-support nodes (e.g., 14–35%) are consistent with increased amino acid substitutions in variable regions (e.g., positions 289–295), which reduce phylogenetic resolution.

Overall, the sequence logo effectively delineates functionally constrained residues that stabilize phylogenetic groupings from variable sites that contribute to sequence divergence and reduced branch support, thereby providing insights into the structural and evolutionary dynamics of the protein family.

Domain verification analysis confirmed the presence of conserved ABC transporter motifs, including ATP-binding and ABC signature motifs, providing additional support for the predicted transporter function of Lr34. These conserved regions likely represent structurally and functionally important domains, such as putative ATP-binding, catalytic, or substrate-interaction sites, that have been maintained under strong purifying selection throughout evolution. Their high degree of conservation across species suggests essential roles in preserving protein structure and function, thereby contributing to the limited sequence variation observed among Lr34-related proteins.

Multiple hydrophobic regions were also identified within the C-terminal half of the protein, consistent with transmembrane helices involved in substrate translocation. Comparison of the partial ABCG transporter sequences from T. monococcum (AOC59458–AOC59466) with Lr34 revealed extensive sequence conservation within these regions, indicating strong evolutionary conservation across wheat species.

In contrast, variable regions (e.g., the third segment) may contribute to functional diversification and environmental adaptation. These sites likely represent flexible regions where sequence divergence occurs between species or strains, potentially modulating protein specificity, interaction capacity, or regulatory properties. Such variability may underlie lineage-specific functional specialization.

Integrating phylogenetic and sequence logo analyses provides a robust framework for identifying functionally important residues within protein families. This combined approach is particularly useful for pinpointing candidate sites for targeted mutagenesis and protein engineering, as conserved residues are likely essential for core activity, whereas variable positions may tolerate substitutions that alter function or specificity without disrupting structural integrity.

Furthermore, conserved motifs often reflect regions involved in shared biochemical functions across homologous proteins, whereas sequence variability outside these motifs contributes to functional plasticity and adaptive potential.

**Figure 4:**
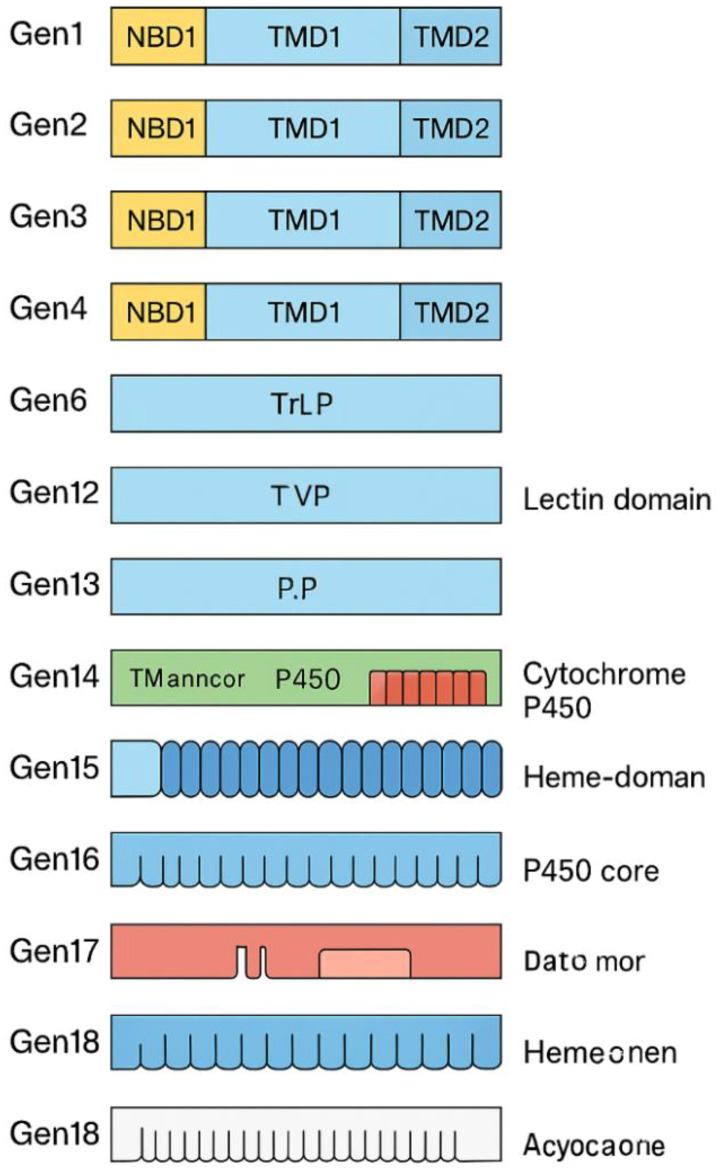
Domain architecture of 18 proteins, with partial datasets excluded due to missing sequence information, highlighting extracellular regions, transmembrane (TM) domains, kinase domains, heme-binding sites, sugar transport motifs, and nucleotide-binding domains (NBDs) characteristic of ABC transporters.

In addition, the domain architecture of wheat proteins is central to understanding their functional complexity (**Figure 4**). Multi-domain proteins enable integration of distinct biochemical activities, such as signal perception and downstream response. For example, proteins containing both kinase and regulatory domains can couple environmental or developmental signals to phosphorylation-based signaling cascades. This modular organization supports coordinated regulation of stress responses, developmental processes, and pathogen defense mechanisms in wheat.

Sequence inspection of the full-length Lr34 protein (ADK62371.1) confirmed the presence of characteristic ATP-binding cassette (ABC) transporter features. The first nucleotide-binding domain (NBD1) contains the highly conserved Walker A/P-loop ATP-binding motif (LGPPGCGKSTLL), while the canonical ABC transporter signature motif (LSGGQ) was identified within the ATPase region. A second ATP-binding cassette motif (GVSGAGKTTLL) was also detected in the C-terminal region, indicating the presence of a second nucleotide-binding domain. These conserved motifs are hallmarks of functional ABC transporters and support the classification of Lr34 as a member of the ABCG transporter family (Kang et al., 2001).

In addition, multiple hydrophobic regions were observed throughout the C-terminal portion of the protein, consistent with transmembrane helices that form the substrate-translocation pathway characteristic of ABC transporters. Comparison of the partial ABCG transporter sequences from *Triticum monococcum* (AOC59458–AOC59466) with Lr34 revealed extensive sequence conservation within the transmembrane region, indicating strong evolutionary conservation across wheat species. Together, these findings provide independent sequence-based support for the transporter architecture predicted by structural modeling.

### Structural modeling

**Figure 5:**
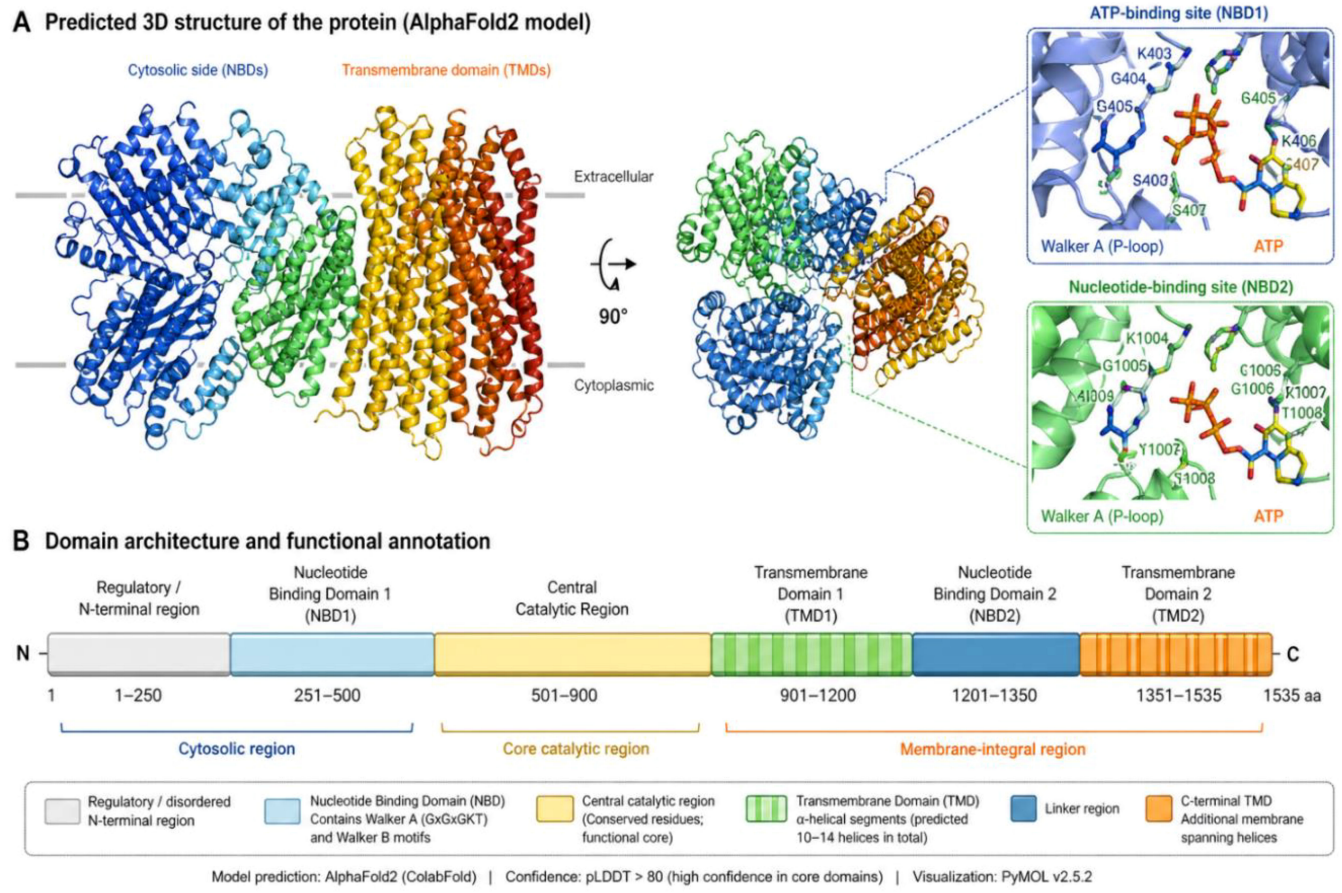
Structural modeling and domain architecture of Leaf Rust Resistance genes in some species of Wheat.

Consistent with the domain verification analysis, AlphaFold2 predicted a canonical ABC transporter architecture comprising two nucleotide-binding domains and multiple transmembrane helices. Consistent with the domain verification analysis, the 3D structure predicted using AlphaFold2 revealed a canonical ABC transporter fold (**Figure 5**) (Kos and Ford, 2009; Higgins and Linton, 2004). The model comprises two cytosolic nucleotide-binding domains (NBDs) and a transmembrane domain formed by tightly packed α-helices embedded within the lipid bilayer. The NBDs adopt globular conformations with high prediction confidence (pLDDT > 80), indicating reliable structural modelling. These domains are positioned on the cytoplasmic side and are spatially arranged to support ATP binding and hydrolysis.

In contrast, the transmembrane region forms a helical bundle consistent with a substrate-translocation pathway across the membrane. The orientation and packing of the helices suggest the formation of a consistent with transport channel, enabling substrate movement through the lipid bilayer (Dean et al., 2001).

Mapping of conserved residues revealed that key amino acids, including glycine (G), lysine (K), and serine/threonine (S/T), are concentrated within the NBDs and catalytic core regions, supporting their role in ATP coordination and hydrolysis. The spatial clustering of these conserved residues further indicates a catalytically competent architecture (Higgins and Linton, 2004).

Integration of sequence conservation with the structural model showed that conserved motifs identified in the sequence analysis correspond to structurally constrained regions within the NBDs. This correspondence reinforces the functional importance of these domains. In plants, ABC transporters are commonly associated with metabolite transport, detoxification, and stress responses. The structural and sequence features observed here therefore suggest that this protein likely participates in similar biological processes in wheat.

## Discussion

The *Lr34* gene is widely recognized as a multi-pathogen resistance gene in wheat, conferring durable, slow-rusting resistance against leaf rust and several other foliar diseases (Krattinger et al., 2009). This study aimed to characterize the molecular diversity and **examine evolutionary relationships** among a set of protein sequences (Gen1 to Gen18) by integrating maximum likelihood phylogenetic analysis with sequence logo based conservation profiling of a defined protein region, following approaches previously described in resistance gene evolution studies (Rinaldo et al., 2016; Jones et al., 1992).

The analyses provide a comparative assessment of sequence diversity among the analyzed Lr34 proteins and identify conserved and variable residues that may be useful for future studies investigating allelic diversity and their potential relevance to wheat breeding (Savadi et al., 2018; Schnippenkoetter et al., 2017).

Phylogenetic analysis revealed clear clustering of sequences, with several internal nodes showing strong statistical support (up to 97%), indicating close evolutionary relationships and high similarity at the protein level. In contrast, other branches exhibited low bootstrap support (14–35%), indicating weaker statistical support and reduced phylogenetic resolution among these groups. These differences may reflect limited phylogenetic signal within the analyzed region or relatively small sequence differences among some sequences.

Consistent with this, sequence logo analysis identified a mixed pattern of conservation and variability within positions 260–301. Several residues, including Y, D, G, A, M, and L, were strongly conserved, suggesting that these positions may be under stronger evolutionary constraint and could contribute to maintaining structural integrity or protein function. In contrast, positions characterized by lower information content and heterogeneous amino acid representation indicate localized sequence variation among the analyzed proteins. Previous studies have also reported that rust resistance genes may exhibit epistatic and complementary interactions, reflecting functional complexity within this gene family (Krattinger et al., 2013; Annan and Huang, 2023).

From an evolutionary perspective, the coexistence of conserved and variable residues is consistent with the evolutionary patterns commonly observed in plant resistance proteins, where conserved positions are generally maintained by purifying selection, while variable sites may contribute to sequence diversification. However, the present analyses do not directly establish the functional significance of individual amino acid substitutions, and experimental validation would be required to determine their effects on Lr34-mediated resistance, consistent with previous reports on resistance gene evolution (Mcintosh, 1992).

In summary, the integration of phylogenetic reconstruction, sequence logo analysis, conserved motif identification, and structural predictions provides a comprehensive computational assessment of sequence conservation, evolutionary relationships, and structural characteristics of Lr34-related proteins. These analyses provide computational evidence supporting the classification of LR34 as an ABC transporter and identify candidate regions that may contribute to protein structure and function. Although experimental validation would provide direct evidence for these proposed functional roles, the primary objective of this study was to conduct a comprehensive bioinformatics investigation; therefore, in planta functional validation was beyond the scope of the present work. Consequently, the findings should be regarded as hypothesis generating rather than definitive evidence of biological function. Future studies employing gene expression analyses, mutant characterization, transgenic approaches, and in planta functional assays will be essential to validate the proposed biological roles and resistance mechanisms (Sugden et al., 2013).

## Data Availability

The datasets presented in this study can be found in online repositories. The names of the repository/repositories and accession number(s) can be found below: https://www.ncbi.nlm.nih.gov/, ADK62371.1 https://www.ncbi.nlm.nih.gov/, ACN41354.1 https://www.ncbi.nlm.nih.gov/, AOC59466.1 https://www.ncbi.nlm.nih.gov/, AOC59465.1 https://www.ncbi.nlm.nih.gov/, AOC59464.1 https://www.ncbi.nlm.nih.gov/, AOC59463.1 https://www.ncbi.nlm.nih.gov/, AOC59462.1 https://www.ncbi.nlm.nih.gov/, AOC59462.1 https://www.ncbi.nlm.nih.gov/, AOC59460.1 https://www.ncbi.nlm.nih.gov/, AOC59459.1 https://www.ncbi.nlm.nih.gov/, AOC59458.1 https://www.ncbi.nlm.nih.gov/, ADK62373.1 https://www.ncbi.nlm.nih.gov/, ADK62372.1 https://www.ncbi.nlm.nih.gov/, ADK62370.1 https://www.ncbi.nlm.nih.gov/, ADK62367.1 https://www.ncbi.nlm.nih.gov/, ADK62366.1 https://www.ncbi.nlm.nih.gov/, ADK62369.1 https://www.ncbi.nlm.nih.gov/, ADK62368.1.
